# Relevant Aspects of Nutritional and Dietary Interventions in Non-Alcoholic Fatty Liver Disease

**DOI:** 10.3390/ijms161025168

**Published:** 2015-10-23

**Authors:** Maria Catalina Hernandez-Rodas, Rodrigo Valenzuela, Luis A. Videla

**Affiliations:** 1 Department of Nutrition, Faculty of Medicine, University of Chile, Santiago 8380453, Chile; E-Mail: cata.hernandezr@gmail.com; 2 Molecular and Clinical Pharmacology Program, Institute of Biomedical Sciences, Faculty of Medicine, University of Chile, Santiago 8380453, Chile; E-Mail: lvidela@med.uchile.cl

**Keywords:** NAFLD, lifestyle, diet, exercise, vitamins, amino acids, prebiotics, polyunsaturated fatty acids, polyphenols, medicinal plants

## Abstract

Non-alcoholic fatty liver disease (NAFLD) is the main cause of liver disease worldwide. NAFLD is linked to circumstances such as type 2 diabetes, insulin resistance, obesity, hyperlipidemia, and hypertension. Since the obesity figures and related comorbidities are increasing, NAFLD has turned into a liver problem that has become progressively more common. Currently, there is no effective drug therapy for NAFLD; therefore, interventions in lifestyles remain the first line of treatment. Bearing in mind that adherence rates to this type of treatment are poor, great efforts are currently focused on finding novel therapeutic agents for the prevention in the development of hepatic steatosis and its progression to nonalcoholic steatohepatitis and cirrhosis. This review presents a compilation of the scientific evidence found in the last years showing the results of interventions in lifestyle, diet, and behavioral therapies and research results in human, animal and cell models. Possible therapeutic agents ranging from supplementation with vitamins, amino acids, prebiotics, probiotics, symbiotics, polyunsaturated fatty acids and polyphenols to interventions with medicinal plants are analyzed.

## 1. Introduction

The burden of nonalcoholic fatty liver disease (NAFLD) has a clinical significance in the health system and is a public health problem affecting about a third of the Western population [1]. NAFLD afflicts 30% of the adult population [2] and the majority of obese individuals [3], making obesity the main promoter disease condition. In the pediatric population, NAFLD has also begun to be a relevant problem in public health due to the etiology and pathogenesis are not fully understood, the significant increase in prevalence and the impact of its progression in level of hepatic dysfunction and associated diseases such as diabetes and cardiovascular diseases [4]. Traditionally, the NAFLD has been considered the hepatic manifestation of the metabolic syndrome. However, recently, researchers indicated that this conventional view of NAFLD is outdated and it has been suggested that NAFLD is a precondition to the development of type 2 diabetes mellitus and metabolic syndrome [5]. Lonardo *et al.* [5] in a systematic review found that in 28 longitudinal studies provided sufficient evidence to consider NAFLD as a risk factor for the emergence of future metabolic syndrome and in 19 longitudinal studies reported that NAFLD precedes the metabolic syndrome and is a risk factor for its development [5]. Liver steatosis is mainly a consequence of excess caloric intake and lack of physical activity, which points to the correction of unhealthy lifestyles as first step to follow in the prevention and handling of NAFLD. When such intervention is inefficient or inadequate, then drug therapy becomes the second strategy; however, the efficacy and safety of the proposed drug treatments for treating NAFLD are still unclear [6].

## 2. Lifestyle Intervention and NAFLD

**Figure 1:**
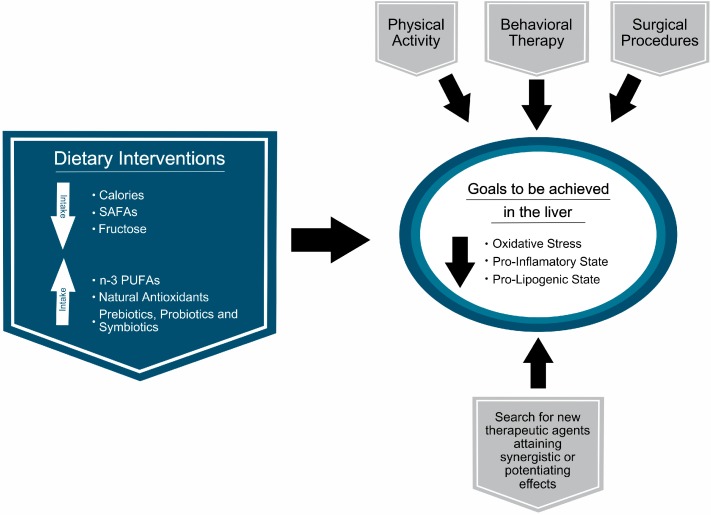
Interventions for the prevention and treatment of non-alcoholic fatty liver disease (NAFLD). *Abbreviations*: n-3 PUFAs, n-3 polyunsaturated fatty acids; SAFAs, saturated fatty acids.

Today, the therapeutic strategies are aimed at reducing the incidence of risk factors involved in the progression of the hepatic disease and comorbidities associated with NAFLD [7]. Nowadays, all the international guidelines report that lifestyle changes that include diet are the only therapeutic approach recommended (Figure 1). As can be observed in Table 1, a variety of human trials and reviews have evaluated the effects of lifestyle interventions in NAFLD. There are limited data on details of how much and how fast weight loss through diet modification must be attained [8], and, besides, extrahepatic and benefits in the liver granted by weight loss are not well explained [9]. The quality and speed of weight loss have been reported to be important, but not explicitly beneficial [10]. In this regard, a moderate weight loss in the same way that physical activity induces a reduction in insulin resistance, and both behaviors are considered as the current therapeutic strategy for patients with NAFLD who are overweight or obese. However, it has been observed that liver biochemistry (alanine aminotransferase (ALT) serum) and the hepatic steatosis share is modified in the presence of dietary treatment, but inflammation and fibrosis are unchanged [9]. Likewise, physical inactivity and type of physical activity are factors that have different effects on the health of the liver and the achievement and maintenance of a healthy body weight. In this regard, vigorous physical exercise reduces insulin resistance, helps maintain weight loss over time and improves hepatic histology. However, mild or moderate exercise intensity does not provide a significant benefit over protection in the development of NAFLD [11]. Similarly, intervention studies looking to increase adherence to the Mediterranean diet and level of physical activity have reported that adherence to the Mediterranean diet is considered a significant predictor of changes in liver fat content in patients with fatty liver, who are non-alcoholic and overweight and that the effect of the diet is gradual and favorable and it is independent of other changes in lifestyle; so the qualitative profile of the intervention from the diet is responsible for the benefits and instead the concurrent weight loss is negligible [12]. Therefore, weight loss and calorie restriction can be a poor approach for the problem of metabolic liver disease, since other factors like the quality of food, lifestyle and exercise, have a significant impact on non-alcoholic fatty liver and these have been less studied.

Since there is no consensus about weight loss in the NAFLD treatment, Ghaemi *et al.* [13] implemented a pilot study that assessed the effects of weight loss on characteristics of NAFLD and associated conditions. For this purpose, 44 NAFLD patients received a diet including a reduction in the daily intake from 1000 to 500 kcal, with a distribution with respect to the total caloric value of 30% fat, 15% protein and 55% carbohydrate for six months. At the end of follow-up period, patients were classified as adherent or non-adherent to treatment according to a weight loss ≥5% or <5% of initial body weight, respectively. After the intervention, 56.8% of patients were classified as adherent group and the 43.2% as non-adherent group, and significant reductions were found in the adherent group in relation to diastolic blood pressure (80.2 to 76.9 mmHg) and the serum levels of total cholesterol (TC), low-density lipoprotein LDL cholesterol (LDL-C), triglycerides (TGs), alanine aminotransferase (ALT), aspartate aminotransferase (AST) and gamma-glutamyl transpeptidase (GGT) activities. These results suggest that reduction in weight is a good therapeutic strategy for obese patients with NAFLD, which reach a weight loss of 9.7% of initial body weight after six months of dietary therapy [13]. Similarly, a systematic review of the impact of non-surgical treatments currently available for liver disease and NASH was conducted in order to determine the metabolic risk in publications, which included a total of 78 randomized trials (30 in nonalcoholic steatohepatitis (NASH) and 40 in NAFLD) [14]. It was found that weight loss was safe, improved the cardiometabolic risk profile, and that a weight loss of ≥7% recovered the histological features of the disease; however, this weight loss was only achieved in less than 50% of patients [14].

The interventions focused on changes in lifestyle are considered a key element for the treatment of NAFLD and metabolic syndrome, and although the optimal strategy has not been developed, and that weight loss and exercise are essential, the long-term sustainability of any intervention is a key factor for this to be successful [15]. While interventions aimed at weight loss are recommended, the reduction in weight by dietary restriction is on average unsustainable in the long term, considering that weight loss usually returns to the initial weight over time [16]. Therefore, appropriate strategies to reduce NAFLD that not only include weight loss are necessary, physical activity being an important factor playing a protective healthy role in NAFLD [17] (Figure 1).

Few studies have demonstrated the efficacy of physical activity, in combination with a diet and weight loss. In a prospective study of 141 individuals with NAFLD who were approached in interventions in lifestyle with mild or moderate intensity, those who increased or maintained their physical activity at a level of 150 min/week or more had greater improvement in liver enzymes, regardless of changes in the weight [18]. In another study of 44 patients who completed a regular exercise program, serum ALT was normalized in 55% of patients, whereas 34% that did not meet the exercise program did not present any standardization [19]. Even small gains in fitness and physical activity can have significant health benefits for patients with NAFLD, as found when practiced training exercise by a very short period (4 weeks) attained decreased liver lipids in obese patients without changes in body weight [20].

However, as the success in changing lifestyle is influenced by personal beliefs and values, it becomes complex to encourage patients to make changes in unhealthy behaviors. Accordingly, behavioral and psychological strategies are considered necessary [6]. Behavioral treatments are global therapeutic approaches that give patients practical tools to achieve their goals of intake and exercise [21]. Few studies have evaluated the effects of behavioral approaches in patients with NAFLD; two out of 10 were controlled trials [22]. These studies compared the effects of Vitamin E and Orlistat against behavioral treatments, and both were in favor of the behavioral approach, with an added effect of Orlistat in weight loss. More recently, intensive interventions of lifestyles in patients with different backgrounds of disease (28 with suspected of fatty liver) were shown to be more effective than the prescription of a dietary standard, both in terms of weight loss and in liver enzymes [23].

Few randomized controlled trials (RCTs) have evaluated the effectiveness of treatments for NAFLD in pediatric population. At present, weight loss by controlling caloric intake, the improvement in the quality of diet and exercise are considered the first line of treatment [24]. An analysis of the current management of pediatric NAFLD showed bariatric surgery and drug treatment with Orlistat and insulin sensitizers are not recommended as first or second line treatment for NAFLD. Thus, interventions focused on changes in lifestyle through diet and exercise remain the first line treatment of NAFLD in children population [5]. Trials have shown that treatments with cysteamine bitartrate, probiotics, polyunsaturated fatty acids (PUFAs) and pentoxifylline have beneficial effects. However, few RCTs with powered statistical that have evaluated the impact of these treatments on histological changes. In the case of vitamin E, it was shown to have beneficial effects and was able to improve liver morphology in children with non-alcoholic steatohepatitis. In conclusion, in pediatric patients the interventions in lifestyle are the first choice of treatment and vitamin E should be considered for children with demonstrated NASH or for those at risk of NASH who have failed to the first choice of treatment. While other therapies show promising results, large RCTs with persuasive endpoints are needed [5].

**Table 1:** Studies on lifestyle interventions in non-alcoholic fatty liver disease (NAFLD).

Intervention	Findings	Reference
Weight loss ≥5% of initial body weight	Significant reduction in systolic blood pressure, total cholesterol, low-density lipoprotein cholesterol, triglycerides, alanine aminotransferase (ALT), aspartate aminotransferase, and γ-glutamyl transferase in the adherent group (weight loss ≥5% of initial body weight)	[13]
Weight loss (≥7%)	Weight loss is safe and improves liver histology and cardiometabolic profile, but it is only achieved in <50% of patients	[16]
Increasing or maintaining the level of physical activity in 150 min/week or more	Greater improvement in levels of liver enzymes, independently of changes in weight	[18]
Complete a regular exercise program	ALT normalization	[19]
Training exercises for 4 weeks	Reduction in liver lipids in obese patients even in the absence of changes in body weight	[20]
Intensive lifestyle interventions	Intensive lifestyle interventions were more effective than the prescription of dietary standard, both in weight loss and in liver enzymes	[23]
Review of the current management of pediatric NAFLD	Lifestyle interventions should be the first line treatment for pediatric NAFLD. Vitamin E could be considered for those with non-alcoholic steatohepatitis (NASH) demonstrated by biopsy or those at risk for NASH where the first line therapy has failed. Other therapies require large RCTs in pediatric population	[5]

## 3. Dietary Interventions

Various changes in dietary intake have occurred in recent years, which are characterized by an increase in energy intake (24%) due to enhancements in the consumption of flour, cereal products, added sugar and fats, and/or in total fat and fruit intake [25]. The use of corn syrup or high fructose as sweeteners in beverages has increased to comprise 41% of the total sweeteners, with added sucralose accounting for 45%, changes that have undoubtedly helped to increase the prevalence of NAFLD, in association with enhanced obesity and fructose intake from soft drinks [26]. Consequently, a number of diet interventions in different models of NAFLD have been evaluated, as shown in Table 2. In Addition, given the high prevalence of NAFLD in adolescents and its close relationship with cardiovascular disease (CVD), it is imperative to implement strategies focused on prevention through diet and changes in lifestyle, and the validation of effective treatment options [27].

### 3.1. Caloric Restriction and Macronutrient Distribution

With the central point that insulin resistance is one of the main problems in NAFLD, a diet with low carbohydrate intake could be considered a reasonable treatment option for these patients (Figure 1). Similarly, studies have reported the benefits of a diet with caloric restriction independent of macronutrients distribution [28], and soy products also have been considered as an important component in the diet for the treatment of NAFLD in the animal model [29], since soy isoflavones increase the antioxidant capacity and reduce hepatic lipid deposits [30]. The latter effect may be related to inhibition of lipogenic transcription factor sterol regulatory element binding protein 1c (SREBP-1c) and activation of peroxisome proliferator-activated receptor alpha (PPAR-α), upregulating fatty acids (FA) oxidation enzymes in the liver [30]. Kani *et al.* [31] evaluated the effects of a diet low in calories and low in carbohydrate containing soy in patients with NAFLD through a RCT of 45 patients with NAFLD who received three different diets: The low calorie diet (restriction of 200–500 calories according to the requirements of each participant and a distribution of 55% of calories from carbohydrates, 15% from proteins and 30% from fat), the low-calorie and low-carbohydrate diet (the same calorie restriction but the distribution was 45% carbohydrates, 35% fat and 25% protein), or a diet low in carbohydrates and in calories, containing soy (the same calorie restriction, the distribution was 45% carbohydrates, 35% fat and 25% protein, but 30 g of soy nut was incorporated instead of 30 g of red meat) for 6 weeks. It was found that changes in both weight and in lipid profile were not significantly different between the 3 groups, but the low-calorie, low-carbohydrate containing soy diet could reduce further the levels of serum ALT, AST, fibrinogen, and of the lipid peroxidation indicator malondialdehyde (MDA), over those achieved by the low-calorie diet [31].

Few studies have evaluated the relationship between protein intake and NAFLD. Protein supplements have offered short-term benefits against hepatic steatosis and lipid profile in sedentary and obese women [32]. In this regard, the research focus has been on the functional properties of soy intake and nitrogen, because soy protein has been shown to be successful in this scenario [33]. In this respect, dietary recommendations in NAFLD patients are 1.000/1.200 kcal/day for overweight women and 1.200/1.600 kcal/day for overweight men. Ideal diet: 50% carbohydrates, 30% lipids (7%–10% saturated fatty acids), and 20% proteins. Diets are developed to provide a calorie deficit of approximately 500 kcal from usual food consumption causing a weight loss of 0.5–1.0 kg/week [34]. Facts interventions with this type of diet (25% of the caloric value from fat (7% saturated fat, 10% monounsaturated fat and 8% polyunsaturated fat), 35% from protein (animal and plant) and 40% from carbohydrates (50% from whole grains, sugar 25 g and 20 g protein/day)) show that the body mass index (BMI), waist circumference, and body fat mass remained relatively stable, whereas high-density lipoprotein HDL cholesterol (HDL-C) increased significantly and TC, LDL-C, VLDL cholesterol (VLDL-C), TGs, AST, GGT, alkaline phosphatase (ALP), fasting blood glucose, and glycosylated hemoglobin (HbA1c) decreased significantly [34]. When stratify patients according to the increase or reduction in BMI, an association between weight loss and liver profit reflected through the ALP and ALT markers was found, and the AST/ALT ratio was observed, with failure to show any changes in patients that increased their weight. Multivariate analysis showed that waist circumference, ferritin, TGs and markers of glucose homeostasis were the parameters most associated with liver enzymes [34].

Studies in an animal model with high-protein diets have also been developed, evaluating whether a high-protein diet prevents the development of steatosis in C57BL/6 male mice with or without pre-existing liver failure, using diets including low-fat or high-fat, low in protein (11% protein) or high in protein (35% protein), high fat/high protein (42% fat and 35% protein), or high fat/low protein (42% fat and 11% protein) for 3 weeks [35]. The results indicate that diets high in protein decreased the hepatic lipid content to ~ 40% of the corresponding low protein diets, high protein diets being more effective in this regard that reducing energy intake by 80%, which were able to reverse the steatosis induced by pre-existing diet. Compared to diets with low protein, mice fed high protein diets showed increased mitochondrial oxidative capacity and elongation of long chain fatty acids (LCFA), a selective enhancement in plasma branched chain amino acids (BCAA) levels, stellate cells diminution, and a trend towards reduced inflammation [35].

### 3.2. Fructose Restriction

The fructose intake in American adolescents (mainly as soft drink) representing the 12% of daily calories, this high fructose intake exceeds current recommendations and is considered as one of the components dietary responsible for promoting NAFLD in this demographic group [36]. In studies of short-term feeding in experimental animals and humans, fructose intake increases the accumulation of fats in the liver and of TGs in plasma due in part to increased *de novo* lipogénesis [37]. In this sense, it has been seen that patients with NAFLD have a fructose intake 2-fold greater than the average intake in control patients, according to population studies, exhibited significant increases in the hepatic mRNA expression of fructokinase and FA synthase, suggesting a high pro-lipogenic potential with consequent ATP depletion that can promote necroinflammation [38]. Also, fructose is involved in oxidative damage through the reduction of antioxidant defense and improvement in the production of reactive oxygen species (ROS) [39]. Both lipid overload and oxidative stress promote the fructose as a triggering factor in the onset and progression of NAFLD [40]. Dyslipidemia, insulin resistance and oxidative damage induced by high fructose intake may contribute to increased risk of CVD that is evident in patients with NAFLD [41]; it makes lack direct evidence demonstrating the benefits of the restriction of fructose in the liver steatosis and CVD. An intervention double-blind, controlled, randomized trial in 24 Hispanic-American adolescents with overweight who had a frequent intake of sweet drinks and a content of liver fat greater than 8% as determined by magnetic resonance spectroscopy, showed that when patients were submitted to take only beverages with fructose or glucose only (33 g of sugar that match the standard amount of sugar in a typical drink) for four weeks, no significant changes in liver fat or body weight in either group were found. However, in the glucose drinking group there was a significant progress in adipose tissue insulin sensitivity, high-sensitivity C-reactive protein (hs-CRP), and in LDL oxidation, suggesting that fructose reduction improves markers of CVD despite the lack of recovery in hepatic steatosis [27].

### 3.3. Mediterranean Diet

The Mediterranean diet has been extensively investigated in terms of benefits with regard to cardiovascular risk reduction and improved insulin sensitivity. However, studies have specifically examined their effects on NAFLD are scarce.

Protective effects have been attributed to the Mediterranean diet because of the high intake of antioxidants; vegetables are the main source of phenolic compounds on this diet. Moreover, polyunsaturated fatty acids of the n-3 series from fish regulate haemostatic factors that induce protection against a variety of chronic diseases, and besides, the olive oil represents a high intake of monounsaturated fatty acids and a good source of phytochemicals, and some protective properties of the Mediterranean diet on human health have been granted to the polyphenols present in wine [42]. A recent meta-analysis showed that n-3 PUFAs found in the Mediterranean diet were beneficial in reducing hepatic steatosis [43]. In order to evaluate whether intervention with Mediterranean diet could improve insulin sensitivity in individuals with NAFLD and reduce steatosis to a greater extent than current dietary recommendations, 12 nondiabetic subjects (six males and six females) with biopsy-proven NAFLD were enrolled for a transverse randomized dietary intervention for 6 weeks. All participants were subjected to the Mediterranean diet and to a control diet (low in fat and high in carbohydrates) in a random order with a period of six weeks of washing between each of the diets. As a result, the weight loss was not different between the two types of diet; there was a significant reduction in relative hepatic steatosis after the Mediterranean diet compared to control diet, with improved insulin sensitivity being observed only with the Mediterranean diet [44]. However, this diet should be further investigated in subjects with NAFLD since the size of the groups evaluated was very small in this investigation.

**Table 2:** Studies on dietary interventions in non-alcoholic fatty liver disease (NAFLD).

Intervention	Model	Conclusions	Reference
Diets restricted in calories and carbohydrates with soy protein addition	Human	Intervention can have beneficial effects on serum levels of liver enzymes, malonaldehyde and fibrinogen in patients with NAFLD	[31]
Low calorie diet rich in proteins	Human	A protein diet is associated with improved lipid profile, glucose homeostasis, and improved liver enzymes in NAFLD, independently of decreases in body mass index (BMI) or in body fat mass	[34]
High protein diet	Animal	The high-protein diet prevents and reverses the steatosis, regardless of fat and carbohydrate intake, and is more efficient than a 20% reduction in energy intake	[35]
Soft drinks with fructose compared to glucose sodas	Human	Reducing fructose improves several important factors to cardiovascular disease, despite the lack of appreciable improvement in hepatic steatosis in overweight adolescents	[27]
Mediterranean diet	Human	The Mediterranean diet reduces hepatic steatosis and improves insulin sensitivity in insulin-resistant people with NAFLD compared to current dietary recommendations, even in the absence of weight loss	[44]

## 4. Therapeutic Agents

As mentioned above, fatty liver is mainly generated from the excessive caloric intake and lack of physical activity, pointing to correction of unhealthy styles as the first line approach in the prevention and treatment of NAFLD, which when this intervention is insufficient, drug therapy becomes a strategic line [6] (Figure 1). Because weight loss has been reported to have a low rate of success in the long term [45], research has focused on the development and validation of new dietary therapies aimed at preventing the hepatic steatosis and its progression to NASH [46]. The challenge for the development of therapies for NASH is related to the complexity of the disease, which is directly associated with to visceral obesity, dyslipidemia, hyperglycemia, insulin resistance, and oxidative stress [47,48]. Effective treatments are needed in order to prevent progression of simple steatosis to chronic liver disease [49], considering that fatty liver disease is a reversible condition which if not treated early can lead to a terminal liver disease [50]. Although the pathophysiology of NASH is still not fully understood and the treatments available are not entirely satisfactory, therapies that limit liver injury and the occurrence of inflammation and fibrosis are particularly attractive for this condition [51]. Currently, it is a great challenge for the pharmaceutical industry to develop a combined therapy that is effective in NAFLD patients exhibiting obesity, insulin resistance, dyslipidemia, and oxidative stress. Therefore, serious efforts have been directed to explore novel therapeutic agents that may be directed to multiple targets [52], natural products extracted from medicinal plants being rich sources of biologically active substances having effects on health benefits and disease prevention in humans [53]. Accordingly, current investigations have focused on herbal extracts and natural products with antihyperlipidemic and hepatoprotective effects against NAFLD [54], particularly potential sources of antioxidants [55] (Table 3).

### 4.1. Amino Acid Supplementation Interventions

#### 4.1.1. Tryptophan

Earlier studies conducted in hens have suggested that supplementation with the amino acid tryptophan (Trp) reduces hepatic lipid accumulation [56]. Recently, the influence of Trp supplementation on NAFLD induced by a fructose-rich diet was studied in C57BL/6J mice, as precursor of serotonin, a regulator of the intestinal motility and permeability [51]. Under these conditions, NAFLD underlying lipid accumulation and increased portal plasma lipopolysaccharide (LPS) concentrations, resulted in derangement of intestinal barrier functions, as evidenced by depressed expression of the tight-junction protein occluding and the serotonin re-uptake transporter (SERT), changes that were attenuated or abolished by Trp. The authors suggested that modulation of the intestinal barrier and the serotonergic system by Trp supplementation may be of importance as a protective mechanism against development of NAFLD in mice [57], although further studies are required to validate this proposal in humans.

#### 4.1.2. Glutamine

In recent years, glutamine was shown to improve hepatic ischemia-reperfusion injury, alcohol-induced liver injury, and gut-derived endotoxemia, promoting resistance to oxidative stress, reducing inflammatory cytokine release, and regulating immune reactions [58]. Assessment of the influence of glutamine on NAFLD induced in rats by a high fat diet (HFD) revealed that hepatic steatosis was accompanied by significant increased liver lipid peroxidation, tumor necrosis factor α (TNF-α) levels, and of p65 NF-κB expression, with concomitant glutathione (GSH) depletion.

Glutamine supplementation reduced the oxidative status of the liver and inhibited NF-κB expression, in association with improvement of hepatic steatosis, suggesting a protective effect of glutamine in NAFLD [58].

#### 4.1.3. l-Carnitine Intervention

l-carnitine plays a critical role in lipid metabolism as it acts as an essential cofactor for β-oxidation of PUFAs by facilitating their transport into the mitochondrial matrix associated with carnitine palmitoyltransferase I (CPT-I) activation, thus converting fat into energy [59]. In recent years, l-carnitine has been proposed as a treatment option for various diseases including liver disease [59]. Using a NASH model mouse subjected to either HFD, HFD plus l-carnitine, or HFD with α-tocopherol, l-carnitine induced an enhancement in the hepatic expression of genes implicated in the transport of long chain PUFAs, mitochondrial β-oxidation, and antioxidant enzymes, with suppression of markers of oxidative stress and inflammatory cytokines in NASH, changes that were similar to those elicited by α-tocopherol. It was concluded that l-carnitine acts as a protective agent to prevent progression of NASH by favoring mitochondrial β-oxidation and redox systems [59].

### 4.2. n-3 Polyunsaturated Fatty Acids (n-3 PUFAs) Interventions

The n-3 PUFAs are crucial structural components of cellular lipids, substrates for the biosynthesis of physiological mediators, and signaling molecules regulating liver lipid metabolism. The latter feature is achieved by (i) transcriptional activation of the expression of enzymes involved in FA oxidation acting as ligands of PPAR-α; and (ii) suppression of *de novo* lipogenesis by down-regulation of SREBP-1c [60]. Therefore, n-3 PUFA depletion in the liver of NAFLD patients favoring FA and TGs formation over FA oxidation [61] points n-3 PUFAs as specific anti-steatotic drugs for NAFLD (Figure 1) [62]. A systematic review by Parker *et al.* [43] on studies pertaining to the effect of n-3 PUFA supplementation in NAFLD patients, including 9 reports and 355 individuals who were administered either n-3 PUFA treatment or placebo, confirmed a significant reduction in hepatic lipid content. Although there was significant heterogeneity between studies, pooled data suggest that supplementation of n-3 PUFAs reduces liver fat, an effect that persists when data from RCTs are analyzed; however, the optimal dose remains to be established. Interestingly, n-3 PUFAs also improved circulating liver functions markers, TGs and TNF-α level, and hepatic microcirculatory function [43]. It was suggested that future designs of RCTs quantifying the magnitude of the effects of n-3 PUFA supplementation on liver fat are necessary [43], which are also important for liver inflammation and fibrosis outcomes [63].

Studies in pediatric patients with NAFLD have also been developed, the most prominent being the RCT of Nobili *et al.* [64] using docosahexaenoic acid (DHA) treatment (250 and 500 mg/day) *versus* placebo in 60 pediatric patients with NAFLD, with evaluation of the changes in the fat liver content by ultrasonography after 6, 12, 18 and 24 months of intervention, and changes in TGs, ALT, BMI, and the homeostatic model assessment (HOMA) index of insulin resistance. Data reported indicate that DHA decreased liver fat content after 6 months of supplementation, an effect that persists up to 24 months and is equally effective at the two dosages studied, when compared with the placebo group. Furthermore, TGs were lower in DHA-treated children than in controls at any time intervention, ALT was lower in groups with 12 months of DHA treatment onwards, and HOMA was lower in the group given 250 mg DHA/day *versus* placebo group at 6 and 12 months [64], in agreement with the positive outcomes reported in adult NAFLD patients.

### 4.3. Vitamin Supplementation Interventions

#### 4.3.1. Niacin

Niacin is the precursor of nicotinamide coenzymes acting either as oxidants (NAD(P)^+^) in catabolic processes or as reductants (NADPH) in anabolic reactions or in the recovery of the reduced form of antioxidant components, thus decreasing oxidative stress [65]. It has been used for the treatment of dyslipidemia and CVD [66], and proposed to prevent hepatic steatosis and delay NASH induced by HFD [67]. This proposal was tested in Sprague-Dawley rats fed either a standard rodent diet, HFD, or HFD containing 0.5% or 1.0% niacin for 4 weeks. Under these conditions, niacin supplementation in the HFD significantly decreased the content of liver fat, liver weight, liver oxidative products, preventing fatty liver [67]. While niacin had no effect on the mRNA expression of enzymes related to FA synthesis and oxidation including acetyl CoA carboxylase 1 (ACC-1), FAS, and CPT-1, and lipogenic transcription factor SREBP-1c, it significantly down-regulated the mRNA and protein expression and the activity of diacylglycerol acyltransferase (DGAT), a key enzyme in triglyceride synthesis, thus in agreement with its receding effect on steatosis [67].

#### 4.3.2. Vitamin E

Natural vitamin E is fat soluble tocopherol comprising eight isomers including four tocopherols and four tocotrienols, RRR-α-tocopherol being the most abundant and with the highest biological activity, which is mainly related to antioxidation by avoid the propagation of free radical reactions at the cell membrane level [68,69]. The potential role of vitamin E in preventing fat infiltration in the liver was assessed in Wistar rats fed either a standard diet (SD), a diet high in cholesterol and saturated fat (HCSF), or a diet high in cholesterol and saturated fat with added of water soluble vitamin E (10 IU/kg/day; HCSF-E) for ten weeks. The results indicate that vitamin E exerted hypolipidemic and hepatoprotective effects, as evidenced by the lower levels of total cholesterol found in HCSF-E treated rats over the HCSF group, in addition to lower serum glutamic oxaloacetic transaminase (SGOT) and steatosis scores at the end of the study compared with the initial values [70]. However, no significant differences between the different experimental groups were observed in relation to blood glucose and serum lipids [70]. In addition, it has been found that when vitamin E is supplied for a period of 2 years to patients with NAFLD, the histological features of the disease improve but, in turn, an increase in insulin resistance and plasma levels of TGs was observed [14].

### 4.4. Interventions with Prebiotics, Probiotics, and Synbiotics

Current evidence suggests that the accumulation of triglycerides in the liver responds not only to obesity, but also, the intestinal microbiota plays a key role in the development of insulin resistance, fatty liver, fibrosis and necroinflammatory score, and thereby becomes an endogenous factor that favors the development of NAFLD [71,72]. The link between the liver-intestinal axis and NAFLD is associated with bacterial overgrowth in the small intestine and increased intestinal permeability [73]. The intestine has a complex array of species of microorganisms, wherein the concentration and type of these microorganisms is mainly influenced by the host genotype and availability nutrient [74]. The liver is susceptible to exposure to intestinal bacterial-derived products through a functional and anatomical connection with the intestinal lumen via the portal vein system [75]. The contribution of the microflora in the progression of NAFLD is given mainly by the improvement in hepatic oxidative stress as a result of increased ethanol production and LPS in the intestinal lumen, and the subsequent release of inflammatory cytokines from the inflammatory cells [76]. High concentrations of cytokines may increase intestinal permeability via disruption of intercellular tight junctions, resulting in progressive inflammation and fibrosis within the liver [77], TNF-α plays a critical role in both insulin resistance and uptake by the liver of inflammatory cells in NAFLD [78]. In recent years, it became clear that a high degree of inflammation due to metabolic endotoxemia has an implication in various diseases, including that induced by high fructose intake that changes the intestinal microbiota and intestinal barrier permeability, resulting in increased bacteria derived LPS [79]. As the various species of the gut microbiota are involved in different intestinal biological functions, such as the defense against colonization by opportunistic pathogens, development of a suitable gut architecture can contribute to immune system homeostasis [80]. In this respect, it has been found that manipulating the enteric flora may represent a key therapeutic strategy in the treatment of NASH. Intake of probiotics (living microorganisms), prebiotics (oligosaccharides), and symbiotics (mixture of probiotics and prebiotics) has been reported by their ability to modify the composition of the microbiota and thereby restore the microbial balance. So, exert benefits for health protection [81] (Figure 1).

Animal model studies have shown that probiotics can reduce the progression of NAFLD. Among these, one study evaluated the effect of supplementation with the mixture VSL Pharmaceuticals, Inc., Ft. Lauderdale, FL, USA (VSL#3); a probiotic containing 450 billion bacteria in various strains (three types of bacteria: *Streptococcus*, *Bifidobacterium* and *Lactobacillus*), to ob/ob mice for a period of four weeks and showed that in response to supplementation was observed a reduction in hepatic fatty acid content, in liver inflammation and, in addition to an improvement in the insulin resistance in the liver [82]. It has also been shown that treatment with probiotics lead to a direct reduction in the release of pro-inflammatory cytokines by a down-regulation in the activity of transcription factor NF-κB [83]. In the model of NASH induced by HFD in rats, the treatment with VSL#3 resulted in a reduction in the expression of markers of lipid peroxidation, TNF-α, inducible nitric oxide synthase (iNOS) and cyclooxygenase 2 (COX-2), when compared with the control group [84]. Similarly, treatment with VSL#3 resulted in a minor insulin resistance in liver and adipose tissue, thus counteracting the development of NASH and atherosclerosis in genetically dyslipidemic ApoE(−/−) mice [85]. Moreover, Ritze *et al.* [86] studied whether supplementation with *Lactobacillus* rhamnosus (LGG) could alleviate experimental NAFLD in C57BL/J6 mice, through administration of fructose via drinking water containing 30% fructose with or without LGG at a concentration of 5 × 10^7^ colony-forming units (cfu) per g body weight. Upon completion of the intervention period, it was found that treatment with LLG generated an increase of beneficial bacteria in the small intestine as well as a restoration in the duodenal tight-junction protein concentration and reduced of portal LPS levels. In addition, attenuation in the hepatic mRNA expression of TNF-α, interleukin-8 (IL-8), and IL-1β, liver fat accumulation, and portal ALT levels were observed in animals fed the high fructose diet plus LGG [86]. Similar studies with *Lactobacillus casei* strain Shirota (LcS) given orally to mice fed a methionine-choline-deficient diet (MCD) that reduces lactic acid bacteria such as *Lactobacillus* and *Bifidobacterium* in feces, increased not only the LcS subgroup but also the lactic acid types, with concomitant suppression of MCD-diet-induced NASH development [87].

In agreement with experimental studies [82], the use of the probiotic VSL#3 in NAFLD patients for 2 to 3 months improved routine liver damage tests and oxidative stress-related indicators, without improvement in pro-inflammatory cytokines, suggesting that manipulation of intestinal flora should be taken into consideration as adjunctive therapy in NAFLD [88]. Two randomized placebo-controlled double-blind studies showed a significant decrease in liver AST with the administration of probiotics in children [89] and in adults [90]. Also, symbiotic studies have been developed in humans, which are included in a recent meta-analysis reporting favorable results in four RCTs, two of which involved the use of symbiotic by co-treatment of probiotics with fructo-oligosaccharides (FOS), the latter prebiotics being potentially promoters of the growth of beneficial bifidobacteria in the intestinal tract [91]. It is noteworthy that one of the previous trials in patients with NASH including liver biopsies showed improvement in liver histology after 6 months of symbiotic treatment containing *Bifidobacterium longum* and FOS [92]. With the intention of defining whether treatment with symbiotics imply greater effectiveness on changes in lifestyle for the treatment of NAFLD, Eslamparast *et al.* [78] designed a RCT with 52 NAFLD patients treated with either a symbiotic or placebo for 28 days, concomitantly with diet and physical activity recommendations. It was found that symbiotic supplementation with lifestyle modification is a better strategy than life style adjustment alone in NAFLD treatment, leading to attenuation of markers of liver damage, inflammation, and fibrosis [78].

### 4.5. Interventions with Polyphenols

Polyphenols are natural compounds produced by plants comprising a heterogeneous group of agents characterized by hydroxylated phenyl moieties. Among them, two types of compounds are distinguished, namely (i) flavonoids containing a common diphenylpropane skeleton (e.g., flavonoids, flavones, flavonols, flavanols, isoflavones, proanthocyanidins, and anthocyanins); and (ii) non flavonoids mainly comprising mono-phenols alcohols (e.g., hydroxytyrosol), or stilbene phenolic acids (e.g., resveratrol) [93]. Several polyphenols have beneficial actions on human health, with potential mechanisms including (i) non-specific antioxidant action due to the existence of a phenol group capable of scavenging free radicals (Figure 1); and (ii) certain mechanisms focused on interactions of particular structural characteristics of polyphenols with proteins or defined membrane domains [94].

#### 4.5.1. Resveratrol, Catechin and Quercetin

The cardioprotective, anti-cancer, and anti-inflammatory properties of resveratrol have been well characterized, a polyphenol that has been reported to present suitable protective effects on the liver against the hepatic lipid accumulation in response to a HFD [95]. The beneficial effects attributed to resveratrol have been awarded mainly to its antioxidant and anti-inflammatory effects that exert protective tissues such as the liver, kidney and brain against a variety of damage caused by oxidative stress and inflammation [96], raising the proposal that resveratrol can be used in the treatment for metabolic disorders including fatty liver disease [97]. In this context, resveratrol was reported to activate sirtuin 1 (SIRT1) with the consequent stimulation of AMP-activated protein kinase (AMPK) [98] through phosphorylation mediated by liver kinase B1 (LKB1) that protects against liver lipid accumulation by down-regulation of FAS expression induced by high glucose [97]. Similarly, resveratrol also blocks the expression of SREBP-1 through the SIRT1/forkhead box O1 (FOXO1) pathway leading to a lipid-lowering effect in HepG2 cells treated with palmitate [99]. A recent randomized, double-blind, placebo-controlled study assessed the scope of resveratrol supplementation in 50 individuals with NAFLD over subjects given placebo for 12 weeks, both groups being subjected to lifestyle improvement. In both groups, the anthropometric measurements, liver enzymes, and degree of steatosis improved. However, resveratrol supplementation was associated with a significant reduction in liver ALT, inflammatory cytokines, NF-κβ activity, serum cytokeratin 18, and grade of hepatic steatosis, compared to placebo-supplemented group. The authors concluded that resveratrol supplementation along with lifestyle modification is a better treatment for NAFLD than lifestyle improvement alone, which is mainly due to attenuation of inflammation and hepatocyte apoptosis [100]. Diminutions in hepatic inflammation and lipogenesis are also observed in HFD fed mice given 30 mg resveratrol/kg/day for 60 days over control values, as evidenced by significant decreases in mRNA expression of either TNF-α, interleukin-6 (IL-6) and NF-κB or the lipogenic factors PPAR-γ, SREBP-1, and ACC-1 [101].

Epidemiological evidence has reported that intake of green tea (Camellia sinensis) may protect against liver injury due to inverse association with lipid profile and serum ALT [102]. While this approach has not yet been confirmed through RCTs in humans, epigallocatechin gallate (EGCG), the main polyphenol catechin in green tea, have been shown to generate a reduction in hepatic lipid accumulation and serum monocyte chemoattractant protein-1 (MCP-1) levels, in a mouse model of diet-induced NASH [103]. Liver protection against HFD-induced NASH in rats was also attained by a green tea extract after 8 weeks supplementation, as shown by an increase in glutathione status related with the inhibition of liver and adipose tissue inflammatory responses mediated by NF-κβ [46].

Quercetin is a flavonoid present in the human diet with a variety of preventive effects in typical human diseases [104], through mechanisms that include a down-regulation in the activation of NF-κB, inducible nitric oxide synthase expression in IL-1β activated rat hepatocytes [105] and also in the improvement in hepatic damage in rats with biliary obstruction [106]. Other significant beneficial effects of quercetin include a reduction in plasma concentrations of oxidized LDL in patients with overweight [107] and a decreased hepatic steatosis induced by Western diet in C57BL/6J mice [108] and attenuation of inflammation and fibrosis in a mouse model of NASH by MCD [51].

#### 4.5.2. Proanthocyanidins and Anthocyanidins

Proanthocyanidins from grape seeds (GSP) are a complex mixture of polyphenolic bioflavonoids having high antioxidant activity, with preventive effects in some forms of cancer and oxidative injury [109]. In a recent study, the effects of GSP and the insulin sensitizer metformin were assessed individually or in combination in a diet-induced NAFLD in Wistar rats subjected to a high fat and high fructose diet (HFFD) [110]. GSP (100 mg/kg/day) and metformin (50 mg/kg/day) were given orally once a day and for the combined treatment, GSP and metformin was administered at 4 h intervals. HFFD resulted in an abnormal plasma lipid profile, with liver inflammation and steatosis, hepatic TGs levels being reduced by 69%, 23%, and 63% after GSP, metformin, and combined treatment, respectively. Accordingly, GSP reduced the mRNA expression of SREBP-1c and increased that of PPAR-α more effectively compared to metformin in HFFD-treated rats; however, no additive effect restoring lipid levels was observed when GSP and metformin were combined [110].

Anthocyanidins (ACNs) are hydrosoluble flavonoids within the polyphenol class, which are responsible for the red, purple, and blue colors of many flowers, cereal grains, fruits, and vegetables [111]. ACNs alleviate hyperglycemia, modulate endothelial function, and reduce inflammation [112], and are able to modulate lipid metabolism and fat deposits in various tissues including the liver [113]. Since the impact of ACNs on NAFLD is not well defined, a literature search grouping experimental *in vitro* and *in vivo* models and human trials was conducted [111]. Although the interpretation of the evidence from *in vitro* studies is hampered by differences in cell models, experimental protocols, and molecular pathways evaluated, most studies are consistent in that ACNs reduced hepatocellular accumulation of lipids by inhibiting lipogenesis and possibly by promoting lipolysis. In addition, interpretation of the data from *in vivo* studies is difficult, due to the large difference in experimental models of NASH used and the utilization of animals exposed to either synthetic ACNs (e.g., Cyanidin-3-*o*-β-glucoside) or to extracts of foods rich in ACNs (e.g., sweet potato, berries, and oranges). Nonetheless, these studies reported an improvement in systemic and hepatic insulin resistance and serum lipids, sometimes related to weight loss and increased PPAR-α activation inducing lipolysis and diminished lipogenesis, thus decreasing hepatic fat content [111]. Finally, a clinical trial enrolling 48 adult borderline hepatitis patients with increased liver enzymes supplemented with 200 mg ANCs of purple sweet potato (PSP) or placebo twice a day for 8 weeks showed that ACNs are associated with reduced levels of liver enzymes, particularly GGT [114]. Although this feature was not associated with liver damage and liver fat was not confirmed by direct imaging, the researchers suggest that the PSP beverage can offer potential activity hepatoprotective against oxidative stress [114]. The final conclusion of the literature search by Valenti *et al.* [111] is that ACNs can prevent the progression of liver damage related to NAFLD by three independent mechanisms, namely, inhibition of lipogenesis by decrease of SREBP-1c, lipolysis promotion by induction of PPAR-α activity, and reduction of oxidative stress, pointing to foods rich in ACNs as a promising strategy for preventing NAFLD and its complications, however future RCTs are needed to test their hepatoprotective efficacy in NAFLD [113].

### 4.6. Medicinal Plants Interventions

For centuries, products made from natural herbs derived from Traditional Chinese Medicine have been used to treat almost all types of diseases in China [115]. Natural products extracted from medicinal plants are rich sources of biologically active substances and have desirable effects on health benefits and disease prevention in humans [53]; therefore, an increasing number of investigations has been focused on extracts of herbs or natural products with anti-hyperlipidemic and hepatoprotectives effects against NAFLD. Considering the above and also that the use of medicinal herbs is becoming increasingly common for handling of NAFLD, Liu *et al.* [50] conducted a systematic review in order to evaluate both beneficial and detrimental effects of them. The study included 77 randomized trials covering 6753 participants with fatty liver disease, the average sample size was 88 participants per test, and 75 different herbal products were evaluated including single herb products, commercially available proprietary medicinal herbs, and combination formulas prescribed by physicians. It was found that (i) six trials showed a statistically significant effect on hepatic B-ultrasound; (ii) four trials showed a significant increase on liver/spleen computed tomography ratio; and (iii) forty two trials showed reduction in AST levels, forty nine trials in ALT, three trials in ALP, and thirty-two in GGT levels in the herbal group. Overall, these findings indicate that herbal medicines may have positive consequences on fatty liver disease, However, there is insufficient evidence to recommend these medicinal herbs for the management of NAFLD because of the high risk of bias and lack of homogeneous data in studies [50].

#### 4.6.1. Tamarindus Indica Linn

At present time, Tamarindus indica Linn is one of the most important resources of plants for supply of foods and materials [52]. Considering that the seed coat of tamarind contains polyphenols including tannins, anthocyanins, and anthocyanidin oligomers, Sasidharan *et al.* [52] evaluated the ameliorative potential of seed coat of Tamarindus indica (ETS) extracts on HFD-induced NAFLD in rats. At dosages of 45, 90, and 180 mg/kg ETS significantly attenuated the pathological changes associated with NAFLD induced by HFD, namely, hepatomegaly, elevated liver lipids and lipid peroxides, serum ALT levels, free fatty acids, and macro and micro hepatic steatosis. In addition, ETS treatment markedly reduced body weight and adiposity, probably acting in part through anti-obesity, insulin sensitizing, and antioxidant mechanisms [52].

#### 4.6.2. Salvia-Nelumbinis Naturalis (SNN)

Salvia-Nelumbinis naturalis (SNN) formulae (initially called Jiangzhi Granula) was designed, in which Salvia as being the principal element and Nelumbinis, Rhizoma Polygoni Cuspidati, Herba Artemisiae Scopariae the ancillary components [115]. In a study of *in vivo* and *in vitro* model the researchers found that intervention with SNN components in HepG2 cells decreased lipid accumulation and in rats the SNN extract improved the steatohepatitis and conferred a normal lipoproteinemia profile in rats fed high-calorie diet, where the effectiveness of the extract SNN to improve liver function and insulin sensitivity was comparable with medications such as simvastatin and pioglitazone [115].

#### 4.6.3. Ostol Treatment

Ostol is the active compound of Cnidium monnieri extract. Ostol has been described by its anti-inflammatory and cytoprotective effects to promote the oxidation of fat. In an animal model of fatty liver in rats, the ostol treatment induced a decrease in fasting glucose levels and hepatic fat content, besides, resulted in improved insulin resistance [116]. Another study reported that treatment with ostol decreased liver fat content by increasing in the hepatic expression of PPAR-α/γ [117]. Similarly, in a model of NASH, ostol treatment led to an increase in the activation of superoxide dismutase (SOD) and decrease in oxidative stress [118]. Nam *et al.* [119] treated rats Sprague-Dawley with HFD plus ostol (20 mg/kg) 5 times a week and found that compared with the group only HFD, HFD plus ostol group showed a significant decrease in intrahepatic fat (39.4% *versus* 21.0%), the expression of SREBP-1c, FAS and intrahepatic stearoyl CoA desaturase-1 (SCD-1) significantly decreased and the expression of PPAR-α was also significantly higher [119].

#### 4.6.4. Sapindus Mukorossi Gaertn

Studies by Chinese have reported Sapindus mukorossi Gaertn skin is rich in saponins and has properties to regulate fat metabolism and to grant protection to the endothelial cells of blood vessels. However, researchs to provide detailed information about the efficacy of Sapindus mukorossi Gaertn in the prevention and treatment of NAFLD are scarce [120]. Peng *et al.* [120] evaluated in a rat model of NAFLD treatment with an alcohol extract of Sapindus mukorossi Gaertn (AESM) in high dosage (0.5 g/kg), moderate dosage (0.1 g/kg) and low dose (0.05 g/kg). The researchers found that high doses of AESM could relieve AST, ALT, TC, triglycerides, LDL-C, GGT, and also raise HDL-C. Also, the morphology of liver tissue and liver cells began to be normal with this treatment [120].

#### 4.6.5. Sasa Borealis (SBS)

The medicinal benefits of Sasa Borealis Bamboo are mainly given by antidiabetic effects in improving insulin secretion as well as for their hypoglycemic and hypolipidemic, anti-obesogenic and antioxidants effects [121]. For Bamboo, the clinical use for the treatment of hypertension, atherosclerosis, cardiovascular disease and cancer has been reported [122]. However, there have been few studies that have investigated the effects of dietary supplementation with extracts of cane Sasa borealis (SBS) in NAFLD. A recent study examined the effect of supplementation with SBS (150 mg/kg/day) in the presence of a HFD for a cycle of action of 5-week in rats and found that the body weight, liver weight, TGs, TC and lipid accumulation in the liver was significantly lower in the HFD plus SBS group compared with only HFD group. Also in the group supplemented with SBS, the transcription factor PPAR-α is increased significantly and conversely SREBP-1c was suppressed in a meaningful way, in addition, supplementation with SBS lead to a significant reduction in hepatic levels of PPAR-γ mRNA, FAS, ACC1, and enzyme diacylglycerol and acyltransferase-2 (DGAT-2) [121].

**Table 3:** Studies on therapeutic agents used in non-alcoholic fatty liver disease (NAFLD).

Intervention	Model	Findings	Ref.
Tryptophan supplementation	Animal	Increased occludin concentrations and reduced ratios liver weight/body weight	[57]
Glutamine supplementation	Animal	Reduced oxidative stress in the liver, inhibition of the expression of p65 NF-κB (nuclear factor kappa-light-chain-enhancer of activated B cells), and hepatic steatosis improvement	[58]
l-carnitine supplementation	Animal	Prevention of NAFLD progression through upregulation of mitochondrial β-oxidation and the redox system	[59]
Docosahexaenoic acid (DHA) supplementation	Human	Improvement of hepatic steatosis in children with NAFLD. Doses of 250 and 500 mg/day appear to be equally effective in reducing liver fat content	[64]
Niacin supplementation	Animal	Decreased liver fat content, liver weight, liver oxidative products, and prevention of fatty liver. Inhibition of mRNA and protein expression and diacylglycerol acyltransferase (DGAT) activity. No effects on mRNA expression of sterol regulatory element binding protein 1c (SREBP-1c), acetylCoA carboxylase 1 (ACC-1), fatty acid synthase (FAS), and carnitinepalmitoil transferase 1 (CPT-1)	[67]
Vitamin E supplementation	Animal	Vitamin E combined with exercise exert hypolipidemic and hepatoprotective effects in the presence of an atherogenic diet	[70]
VSL mixture (*Streptococcus*, *Bifidobacterium*, *Lactobacillus*)	Animal	Reduction in total fatty acid content in the liver and in hepatic inflammation, with improvement of hepatic insulin sensitivity	[82]
Probiotic treatment	Animal	Down-regulation of the activity of the transcription factor NF-κB	[83]
*Lactobacillus* rhamnosus treatment	Animal	Attenuation of fat accumulation in the liver and in the concentration of portal alanine aminotransferase (ALT)	[86]
*Lactobacillus* casein cepa Shirota treatment	Animal	Suppression in NASH development, reduced serum concentrations of lipopolysaccharide (LPS), inhibition of liver inflammation or fibrosis, and diminished inflammation of the colon	[87]
Supplementation with synbiotic, probiotic and prebiotic cultures along with recommendations on healthy lifestyles	Human	The synbiotic supplementation combined with changes in lifestyle is greater than just changes in lifestyle alone for the treatment of NASH	[78]
Resveratrol supplementation	Human	Resveratrol supplementation together with changes in lifestyle is more effective than just changes in lifestyle alone. This is at least partially due to attenuation of inflammatory markers and hepatocellular apoptosis	[100]
Resveratrol supplementation	Animal	Improvement in lipid metabolism and decreased the pro-inflammatory profile of NAFLD in the liver of mice with diet-induced obesity	[101]
Green tea extract	Animal	Higher glutathione levels, lower protein and mRNA contents of inflammatory cytokines, and lower DNA binding activity of NF-κB in liver and adipose tissue of mice supplemented with a green tea extract 2%	[46]
Quercetin treatment	Animal	Total or partial prevention of hepatic steatosis, inflammatory cell accumulation, oxidative stress, and fibrosis caused by the a methionine-choline deficient (MCD)	[51]
Proanthocyanidins from grape seed (GSP) plus metformin	Animal	Improvement of lipid metabolism, but the effects were not additive to normalize lipid levels	[110]
Review of interventions with anthocyanidins (ACNs) in NAFLD patients	Human	Foods rich in ACNs may be promising for prevention of NAFLD and its complications. However, further studies are required	[111]
Seed tamarindus indica	Animal	Intervention has a therapeutic potential against NAFLD, acting in part through insulin sensitization, antioxidant, and anti-obesity mechanisms	[52]
Ostol treatment	Animal	Decreased intrahepatic fat content and in the expression of SREBP-1c, FAS and stearoyl-CoA desaturase-1 (SCD-1), with increased expression of peroxisome proliferator activated receptor α (PPAR-α)	[119]
Sapindus alcohol extract mukorossi Gaertn supplementation	Animal	Regulation of the level of blood fat and improvement in the pathological changes in liver tissue in a rat model of NAFLD	[120]
Sasa borealis (SBS) supplementation	Animal	Improvement in cholesterol metabolism, decreased lipogenesis, and increased oxidation of lipids in rats with high-fat diet (HFD)-induced hepatic steatosis	[121]

#### 4.6.6. Silimarin

Milk thistle has been known for over 2000 years as a herbal medicinal that has been traditionally used for a variety of pathologies. It has been particularly used for handling to diseases related to the liver and gallbladder. Silibum marianum (Latin term for the plant) and its seeds are rich in a variety of natural compounds called flavonolignans. Silimarin is known as a mixture of these compounds, which is extracted after being processed with ethanol, methanol and acetone and contains mainly silibin A, silibin B, taxofolin, isosilibin A, isolsilibin B, silichristin A, silidianin, and other compounds in smaller concentrationes. Apart from its use in liver and gallbladder disorders, milk thistle has recently gained attention due to its hypoglycemic and hypolipidemic properties [123]. Loguercio *et al.* [124] carried out a multicenter, phase III, doubled-blind critical trial to asses RA (comprises the silybin phytosome complex (silybin plus phosphatidylcholine) coformulated with vitamin E) in individuals with NAFLD histologically documented. The participants were distributed (1:1) to receive active treatment (RA; active components: silybin 94 mg, phosphatidylcholine 194 mg, vitamin E acetate 50% (α-tocopherol 30 mg) 89.28 mg) or placebo (P; extrawhite saccharine replacing active components) with a daily dose of 2 times orally and for a period of 12 consecutive months, and the authors found that patients treated with RA showed relief in values of transaminases (AST, ALT) and GGT, insulin resistance and different histological features of the liver [124]. Similarly, a recent study compare the metabolic effects of the Mediterranean diet *versus* the diet associated with silybin, phosphatidylcholine and vitamin E complex (RE complex) in overweight patients with NAFLD and reported that the treatment for six months with the Mediterranean diet and the RE complex, exhibited improvement not only in anthropometric parameters (reduction in BMI and waits circumference) but also in insulin resistance and hepatic fat accumulation [125].

### 4.7. Miscellaneous Therapeutic Agents’ Interventions in NAFLD: Astaxanthin, Cinnamon, and Coffee

Astaxanthin (ASTX) is a xanthophyll carotenoid this primarily in marine animals, among them in salmon and crustaceans [126], which is a potent antioxidant acting as a free radical scavenger including ROS [127] and peroxyl radicals, thus protecting PUFAs in biological membranes from lipid peroxidation [128]. Seeking to define an effective dose of dietary treatment with ASTX to address metabolic dysfunctions, male C57BL/6J mice were fed a HFD (35%) and were treated with 0, 0.003%, 0.01% or 0.03% of ASTX (*w*/*w*) for 12 weeks [129]. At the highest dosage used, ASTX significantly decreased plasma TGs, AST, and ALT concentrations, and increased expression of endogenous antioxidants genes in liver was observed, with lower sensitivity of isolated splenocytes to LPS stimulation, thereby suggesting that ASTX can have a role in preventing of obesity-associated metabolic disturbances and inflammation [129].

Askari *et al.* [130] designed a RCT to evaluate the effects of cinnamon supplementation in patients with NAFLD, involving 55 patients with NAFLD randomized supplemented either with 2 cinnamon capsules (each capsule containing 750 mg of cinnamon) or placebo capsule daily for 12 weeks, and all patients were instructed to implement a balanced diet and physical activity. Under these conditions, the treatment group exhibited significantly decreased HOMA, fasting blood glucose, TC, LDL-C, TGs, ALT, AST, GGT, hs-CRP, however, the serum levels of HDL-C and in both groups remained unaltered [130].

The coffee is regarded as the most consumed beverage worldwide. A recent large prospective study showed that consumption of pure and decaffeinated coffee is associated with decreased all-cause death [131]. Similarly, it has been reported that the intake of coffee reduces the risk of advanced liver disease and complications associated with this [132], and equally of hepatocellular carcinoma independent of the etiology [133]. However, despite the above benefits, the molecular mechanisms that contribute to the protective effect of coffee are not well clarified. In this regard, a study evaluated the effects of the administration of decaffeinated espresso coffee *versus* placebo in rats fed with HFD and changes in the proteomic profile of the liver. It was found that rats receiving HFD plus placebo developed periacinar steatosis, lobular inflammation, and average fibrosis; while those receiving HFD plus coffee exhibited only average steatosis. Coffee consumption increased the hepatic expression of chaperones of the endoplasmic reticulum and induced the expression of master regulators of redox state. In addition, coffee intake was associated with decreased expression of the α-subunit flavoprotein electron transfer, an element of the mitochondrial respiratory chain related with *de novo* lipogénesis [134].

## 5. Conclusions and Projections

Currently, an effective pharmacological therapy for the NAFLD treatment is not available. Lifestyle interventions involving diet and exercise remain the first line treatment (Figure 1), however, the weight loss long term has a low success rate as well as dietary restrictions adherence. This situation has prompted the exploration of new therapeutic agents for the prevention of hepatic steatosis and the progression of the disease. Scientific evidence of potential therapeutic agents remains lacking, partly because of the lack of clinical trials with based on evidence of liver histopathology data, but also due to the fact that NAFLD is a multifactorial disease involving deep and complex metabolic changes in the liver, which are in close relationship with other tissues such as adipose tissue and skeletal muscle, making the possibility of successfully respond to monotherapy unlikely. This situation points to the need for new therapeutic approaches considering the assessment of the effectiveness of combined bioactive compounds that have proven hepatoprotective actions, in order to find possible additive or potentiating effects in the prevention and treatment of NAFLD. In addition, it would be of importance to consider the effects of therapeutic agents in conjunction with other non-pharmacological therapies, such as those focused on behavioral therapies and surgical procedures, to evaluate the usefulness of complementary mechanisms of actions on NAFLD outcomes.
